# Numerical study of forces acting on the drum cutting coal with gangue

**DOI:** 10.1371/journal.pone.0296624

**Published:** 2024-02-09

**Authors:** Hongmei Liu, Han Zhang

**Affiliations:** College of mechanical engineering, Liaoning Technical University, Fuxin, China; University of Sharjah, UNITED ARAB EMIRATES

## Abstract

A coal model containing gangue was established by discrete elements to study the forces occurring when a shearer cuts coal, and a rigid-flexible coupled shearer section model was established in RecurDyn; the drum cutting process was simulated through bidirectional coupling. The dynamic distribution of the force chain was obtained when cutter teeth cut the coal rock. During the cutting process, the coal rock failure is caused by normal and tangential forces, with the latter having a major role. The ratio of the average tangential force to normal force was 1.34~1.79. When the rock is in the middle of the coal seam, the force (including normal and tangential forces) on the rock is the highest. Moreover, the force on the rock-coal interface is greater than that on the coal-rock interface. The results show that the force on the coal decreases with the increases in rotation speed. In contrast, the number of broken bonds increases. Further, the number of broken bonds and the force of coal increased nonlinearly with traction speed. Finally, the increase of tooth mounting angle decreased the force on the coal rock and the number of broken bonds, followed by an increase. As such, this study provides a reference for further theoretical research on forces in coal cutting.

## 1 Introduction

The shearer design is based on calculating cutting forces acting on it. Evans et al. studied coal and rock breakage caused by the tensile force when the cutting tool cuts into coal. The authors obtained the rock-breaking model of coal during picking, which is widely used in the design of mining machinery in Europe and America [1, 2]. Nishimatsu et al. established the mechanical rock and coal cutting model based on the Coulomb-Mohr theory. The authors pointed out that coal and rock cutting and crushing, according to the Coulomb-Mohr criterion, is mainly caused by shear force [3]. Niu et al. proposed that coal rock is broken along the bedding and joint of the coal body under the action of cutting tools. The authors obtained the rock-cutting model and cutting force expression formula [4, 5]. Zhang et al. used the finite element method to explore coal and rock cutting. The results showed that shear force was the main cause of coal and rock failure [6]. Wang et al. experimentally investigated coal cutting with cutter teeth. The author found that the failure of coal is the result of shear force and tensile stress, where shear forces play a major role [7]. Chen et al. used the discrete element method (DEM) to study coal and rock cutting and found that coal and rock breaking is the result of tensile and shear stresses [8]. Wang et al. employed FLAC 3D (Fast Lagrangian Analysis of Continua) software to study the deformation and failure process of coal under the action of cutting teeth. The authors found that coal failure results from the combined action of tension and shear, where the shear failure is the main failure form [9]. Wang et al. experimentally investigated coal cutting with cutter teeth, concluding that both tensile and shear stresses cause coal failure.

Liu et al. designed a coal-cutting test bed, obtaining the pick cutting force, compressive strength, carbide tip diameter, and cutting depth of the drum. The results show that the cutting [10] force increases linearly with compressive strength [11]. Luo et al. experimentally obtained the relationship between cutting force and coal compressive strength, pick tooth cone angle, drum speed, and traction speed. The cutting load increases with cutting thickness and tooth cone angle and decreases with an increase in drum speed [12]. Luo et al. created a coal-cutting test bed to determine the influence of the axial force on the structural parameters of the drum for coal and rock cutting. The results show that the axial force fluctuation caused by coal cutting is relatively small when the cone angle is 90° [13]. Serdar Yasar compared the experimental maximum cutting force values with their theoretical counterparts through pick-cutting tests [14]. Barker et al. found that the number of fragments increased with the cutting depth and spacing. Chisel-type picks are more effective than pointed picks regarding the best spacing-to-depth ratio [15]. Liu et al. created a coal-cutting test bed and obtained the relationship between the cutting force and compressive strength of coal, the carbide tip diameter, and the cutting depth [16]. Zhao used ABAQUS to study the relationship between the drum force and pick mounting angles–assuming the tangential installation angle of 50° yielded the smallest lateral force on the pick, resulting in the best cutting performance [17]. A cutting dynamics simulation was carried out using LS-DYNA, and the mean force error between the model and the prototype theoretical value was 4.95%, with a peak force error of 16.2% [18].

The DEM is widely used for simulating drum cutting coal. Van Wyk used the DEM to numerically simulate the tool forces in rock-cutting tests [19]. Rojek used the DEM to simulate the rock-cutting process [20]. Bao studied the variations of the coal wall and the force acting on the shearer using DEM [21]. MAO J used DEM to study the forces acting on the shearer drum and its performance, obtaining the optimal speed, traction speed, and cutting depth [22]. Estay D A used the DEM to simulate the rock fracture problem, determining the failure mechanism [23]. Okan Su used the discrete element software PFC to simulate the uniform linear cutting process of the pick; the theoretical simulation results were consistent [24]. McBride et al. used the discrete element software to simulate the vertical screw conveyor, and the effects of speed, blade clearance, and material parameters on the conveying effect were obtained [25]. Wu et al. used PFC3D to simulate the process of the pick breaking the coal, obtaining the pick force, and the relationship between said force and the cutting thickness [26]. Bao et al. simulated the coal drum cutting process via the discrete element software; the variations in the coal wall and the drum force were obtained during the double drum shearer operation [21]. Mao et al. used the DEM to study the dynamic process of coal rock cutting using the coal mining machine. The influence of cutting angles on the coal loading rate, cutting resistance, and the shearer cutting energy consumption were studied [27]. Liu used the DEM to establish a discrete element simulation model of the shearer cutting section, analyzing the relationship between the drum speed, the traction speed, and the coal loading rate [28]. Lastly, Zhang et al. used DEM to study the dynamic process of coal cutting using picks, obtaining the effect of the cutting thickness on the cutting force [29]. The abovementioned investigations demonstrate that many researchers use DEM to study coal and rock drum cutting.

This study established a rigid-flexible coupling shear part model, with a coal model containing gangue being established through discrete elements. The two-way coupling of DEM and multi-body dynamics (MBD) collaborative method was used to study the coal failure force. The influences of drum and traction speeds on the force were analyzed. This paper provides a method for studying the coal force for teeth that contain gangue, providing a reference for the theoretical study of coal cutting.

## 2 Discrete element theory analysis

**Fig 1:**
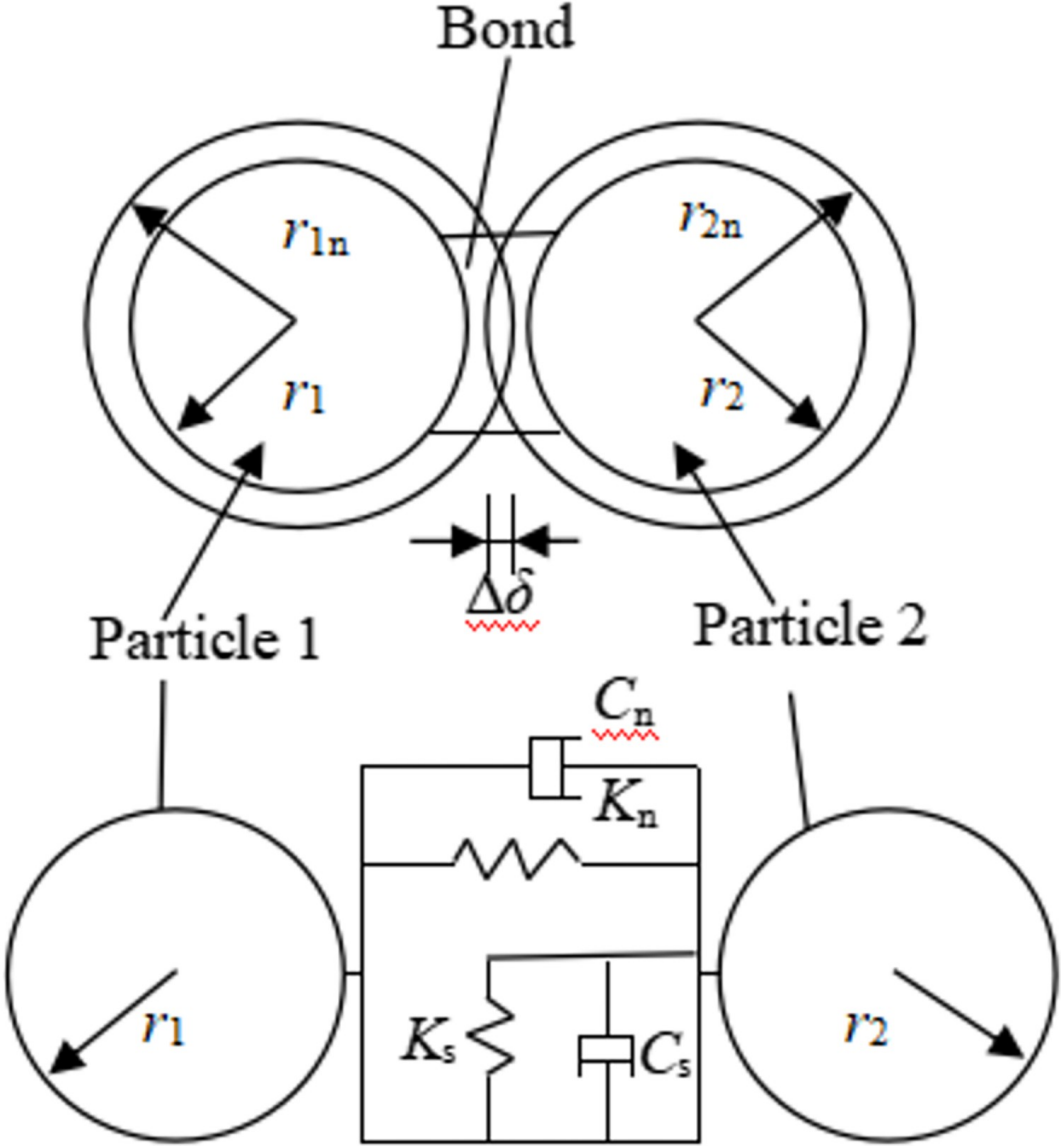
Bonding contact model.


According to Hertz-Medlin bonding theory [30, 31], coal and rock particles are connected by bonds, as shown in Fig 1. The bonding model can transmit forces and moments between contacting particles. The force includes normal and tangential forces, and the moment includes normal and tangential moments, which are calculated via Eqs (1)—(4).

$$\delta F_{n}=-v_{n}K_{n}A\delta t,$$ (1)

$$\delta F_{s}=-v_{s}K_{s}A\delta t,$$ (2)

$$\delta T_{n}=-\omega_{n}K_{s}J\delta t,$$ (3)

$$\delta T_{s}=-\omega_{s}K_{n}\frac{J}{2}\delta t,$$ (4)

where *F*_n_ and *F*_s_ are the normal and tangential particle bonding forces, respectively, N; *T*_n_ and *T*_s_ are the normal and tangential particle bonding moments, respectively, N·m; *A* is the contact area, *A* = *πR*^2^, m^2^; *R* is the bonding radius, $R=\sqrt{r_{1n}r_{2n}}$, m; *r*_1n_ and *r*_2n_ are the contact radius of particles 1 and 2, m; *K*_n_ and *K*_s_ are the normal and tangential contact stiffness of particles, N/m^3^; *J* is the moment of inertia, $J=\frac{1}{2}\pi R^{4}$, m^4^; *v*_n_ and *v*_s_ are the normal and tangential velocities of particles, m/s; *ω*_n_ and *ω*_s_ are the normal and tangential angular velocities of the particles, rad/s; *δt* is the time step, s.

The particle bonds can withstand some stretching and shearing effects. The bond is broken once the force between particles exceeds its strength failure range, and coal particles fall off from the coal. The breaking conditions are given as follows:

$$\sigma_{\max}<\frac{-F_{n}}{A}+\frac{2T_{n}}{J}R,$$ (5)

$$\tau_{\max}<\frac{-F_{s}}{A}+\frac{2T_{s}}{J}R,$$ (6)

where *σ*_max_ and *τ*_max_ are normal and tangential bond failure strengths, respectively, Pa;

Hertz-Medlin bonding model is especially suitable for rocks, coals, and concrete structural models. Therefore, this model is applied to calculate the bonding fracture and breakage between coal and rock particles. The bonding force between particles can disappear under the action of external load, achieving the crushing effect.

## 3 Two-way coupling simulation of discrete element and multi-body dynamics for drum cutting coal

### 3.1. 3D modeling of the shearing section

**Fig 2:**
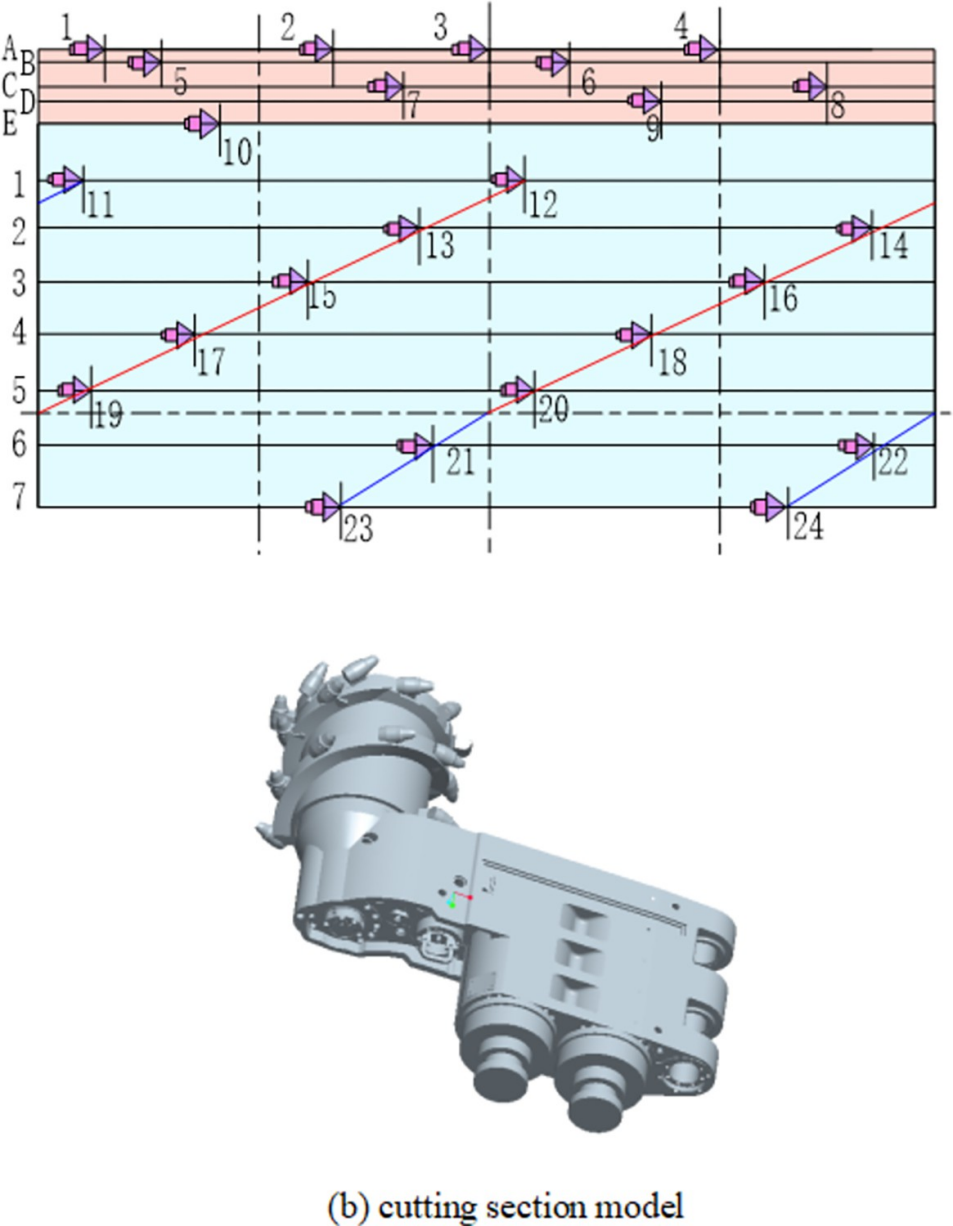
Arrangement of drum picks and cutting section model. (a) drum pick arrangement. (b) cutting section model.

In this study, the MG2×55/250-BWD type shearer was used. The drum pick arrangement is shown in Fig 2A, along with the pick designations. The drum diameter is 800 mm, while the drum hub diameter is 465 mm. Teeth inclination angles from Sections A to D were 15°, 12°, 8°, and 3°, respectively, while the remaining teeth inclination angles were equal to 0°. The pick angles from Section A to Section D were 47°, 35°, 20°, and 12°, respectively, while the remaining pick angles were equal to 0°. Corresponding pick axial heights were 630 mm, 612 mm, 580 mm, 562 mm, 534 mm, 464 mm, 400 mm, 334 mm, 266 mm, 196 mm, 124 mm, and 50 mm, respectively. Finally, the drum and cutting section models were created based on the shearer design using PRO/E (see Fig 2B).

### 3.2. A rigid-flexible coupling dynamic model of the shearer section

The three-dimensional (3D) solid model of the shearer section was imported into RecurDyn, and the density was per material part. According to the relationship between cutting part segments, a fixed pair or rotating connections defined the relative movement between the two [32, 33]. The gear pair was defined as the rotating pair with the addition of physical contact to achieve the gear rotation. According to the Hertz contact theory [34], the contact stiffness between two gears is calculated using Eqs (7)—(9):

$$\frac{1}{R}=\frac{1}{R_{1}}\pm \frac{1}{R_{2}},$$ (7)

$$\frac{1}{E}=\frac{\left(1-\upsilon_{1}^{2}\right)}{E_{1}}+\frac{\left(1-\upsilon_{2}^{2}\right)}{E_{2}},$$ (8)

$$K=\frac{4}{3}R^{1/2}E,$$ (9)

where "+" denotes external gearing and "−" denotes internal gearing; *R*_1_ and *R*_2_ are the pitch circle radii of gears 1 and 2, mm; *R* is the equivalent radius, mm; *υ*_1_ and *υ*_2_ are the corresponding Poisson ratios; and *E*_1_ and *E*_2_ are the Young moduli of gears, N/mm^2^.

The RecurDyn flexible module was used to create the finite element pick grid; the 10-node tetrahedral elements were used. The tooth material was 42CrMo [35], while the Square head, blade, end plate, and barrel body were made of 16Mn steel. The elastic modulus of 42CrMo is 2.125×10^5^ MPa, Poisson’s ratio is 0.3, and its density is 7.85×10^3^kg/m^3^. The elastic modulus of steel is 2.178×10^5^ MPa, Poisson’s ratio is 0.3, and the density is 7.85×10^3^kg/m^3^.

**Fig 3:**
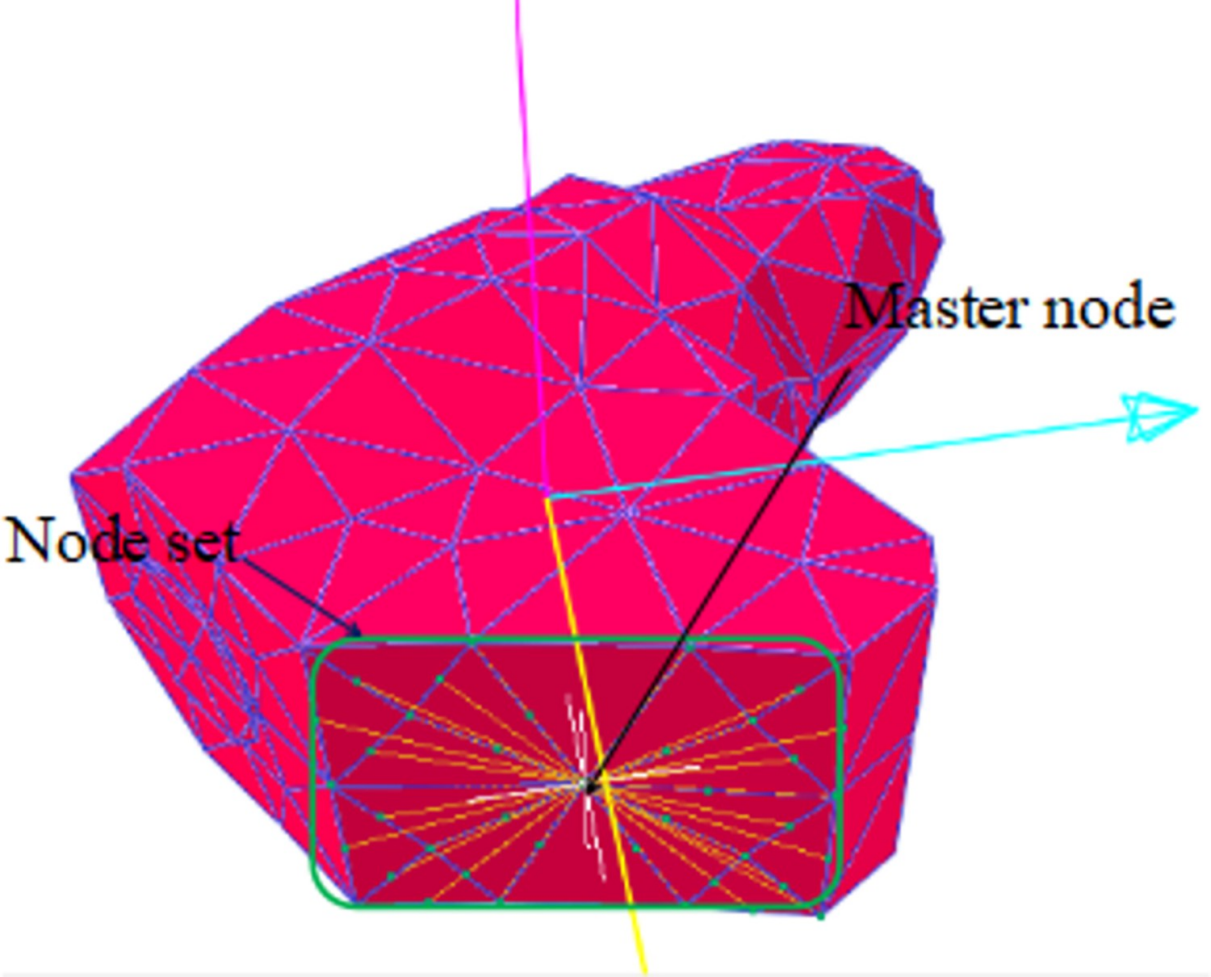
The flexible pick element.

A two-way coupling between the EDEM and RecurDyn was achieved by importing components into EDEM. A wall was generated as a path set using each flexible cutting pick, while the picks were generated as walls. The remaining drum components, such as the end plate, blades, and barrel body, were directly generated as walls. The node set was generated to connect the flexible pick and the drum blade or the end disc–the picks and the drum were in contact with the plane. The master node was selected in a way that generated a node-set and master node form rigid element. A fixed constraint was selected when defining the pick and blade constraints; the fixed constraint point of action was selected as the master node. The flexible pick element is shown in Fig 3.

### 3.3. Discrete element model of drum cutting coal rock

#### 3.3.1. Discrete element model of the coal rock

The coal and rock samples were obtained from Yangcun Mine. Samples were tested [34, 36], and their densities, tensile strengths, compressive strengths, Young moduli, Poisson ratios, cohesion, and internal friction angles were obtained. The obtained parameters are given in Table 1.

**Table 1:** The coal rock parameters.

	Density	Tensile	Compressive	Elastic	Poisson’s	Cohesion	Internal friction	Ruggedness
		strength	strength	modulus	ratio		angle	factor
	/Kg/m^3^	/MPa	/MPa	/MPa		/MPa	/°	
Coal	1 466.92	2.38	15.43	3940	0.33	4.5	38	1.4
Rock	2 455.80	6.22	34.62	20960	0.16	14.2	34	3.5

The Hertz-Mindlin model with bonding contact was used. The coal particles were non-uniform, spherical, and randomly distributed. Their base radius was 12 mm, with the maximum and minimum distributions being 1.5 and 0.5 times the base radius [36]. The Hertz-Medlin bonding contact model bonded the coal and rock particles. In contrast, the coal rock model was obtained via EDEM. The particle contact model parameters are shown in Table 2 [30, 36].

**Table 2:** Particle contact model parameters.

	Normal stiffness	Tangential stiffness	Normal stress	Tangential stress
	/N·m^-3^	/N·m^-3^	/MPa	/MPa
Coal–coal	1.13×10^8^	9.01×10^7^	8.32	2.36
Rock–rock	1.98×10^8^	1.59×10^8^	26.4	13.30
Coal–rock	1.44×10^8^	1.15×10^8^	17.00	7.40

#### 3.3.2. The DEM drum model

The generated drum model wall was imported into EDEM through EDEM and RecurDyn interfaces, with meters (m) being used as a unit of length. The shear modulus, Poisson ratio, and drum density were set according to the modeled material. This enabled the authors to create the discrete-element simulation model of drum cutting coal. The dynamic friction coefficients, static friction coefficients, and recovery coefficients [34, 36] between the coal, drum, and rock are shown in Table 3.

**Table 3:** Coal rock particle parameters.

	Dynamic friction coefficient	Static friction coefficient	Recovery coefficient
Coal–coal	0.05	0.8	0.5
Rock–rock	0.05	0.7	0.5
Coal–rock	0.05	0.75	0.5
Coal–drum	0.01	0.5	0.5
Rock–drum	0.01	0.6	0.5

### 3.4. Bidirectional coupling setting and simulation

The time step is 20% to 40% of the Rayleigh time step. The Rayleigh time step [37] is determined via Eq (10).

**Fig 4:**
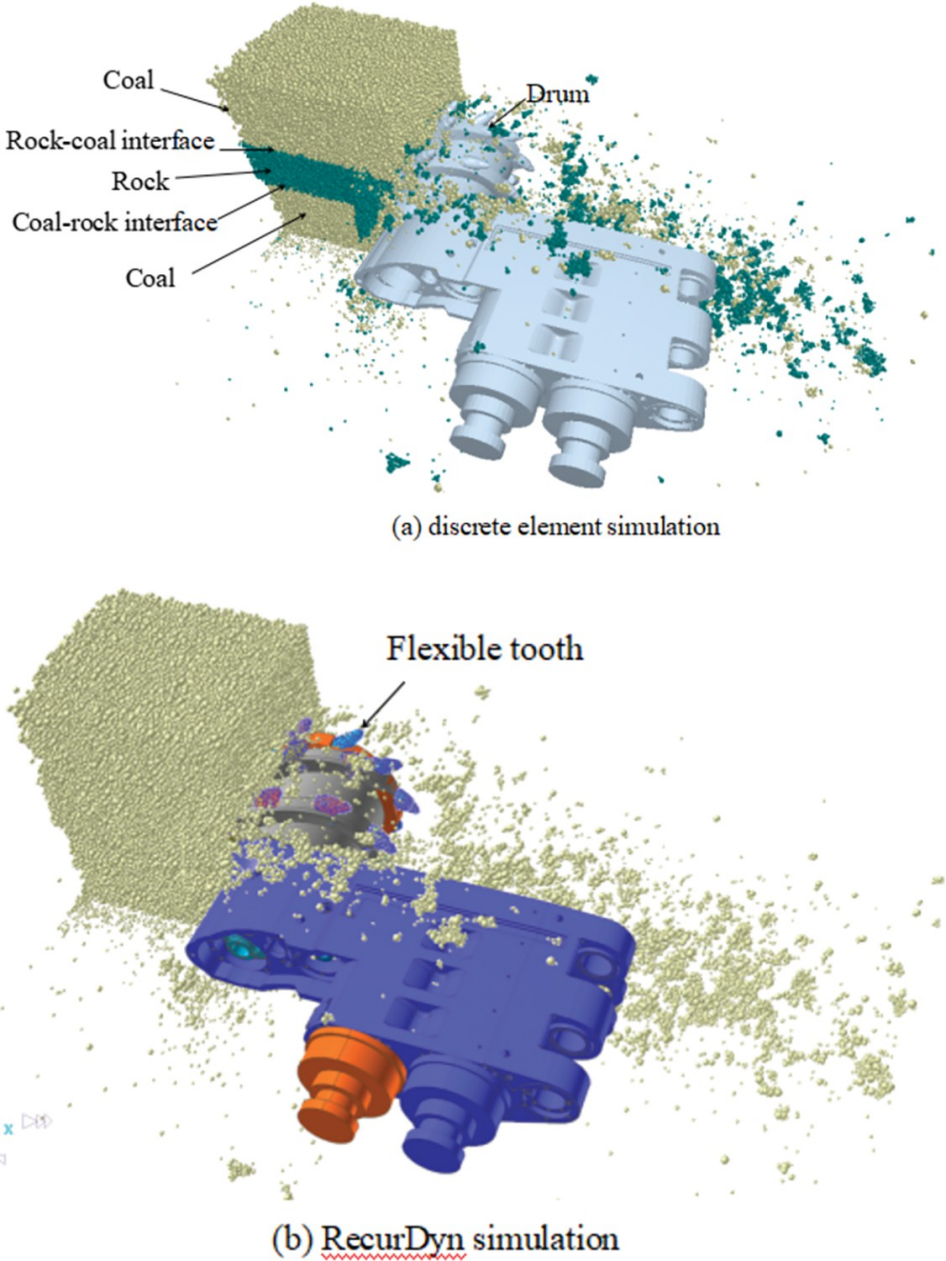
Bidirectional coupling simulation. (a) discrete element simulation. (b) RecurDyn simulation.

TR=πr(ρG)12(0.613μ+0.8766)−1, (10)

where *ρ* is the density of the particles, kg/m^3^; *r* is the radius of coal particles, m; *μ* is Poisson’s ratio; *G* is shear modulus, Pa. The time step is 20% Rayleigh time step, i.e., 5.45×10^−6^ s, to ensure the stability of the EDEM simulation. The target storage time interval was 0.01 s, and the grid size was five times the minimum particle radius. The drive was included in the motor output shaft using RecurDyn, and the step function was used to simulate it. For the motor speed of 159.588 rad/s, the corresponding drum speed was 90 r/min. The traction speed was set to 4 m/min, and the simulation time was 5 s. Couplings were created in discrete element and RecurDyn environments, allowing the authors to conduct a two-way coupling simulation. The discrete element simulation is shown in Fig 4A, while the RecurDyn simulation is shown in Fig 4B.

## Analysis of simulation results

### 4.1 Distribution of force chains

**Fig 5:**
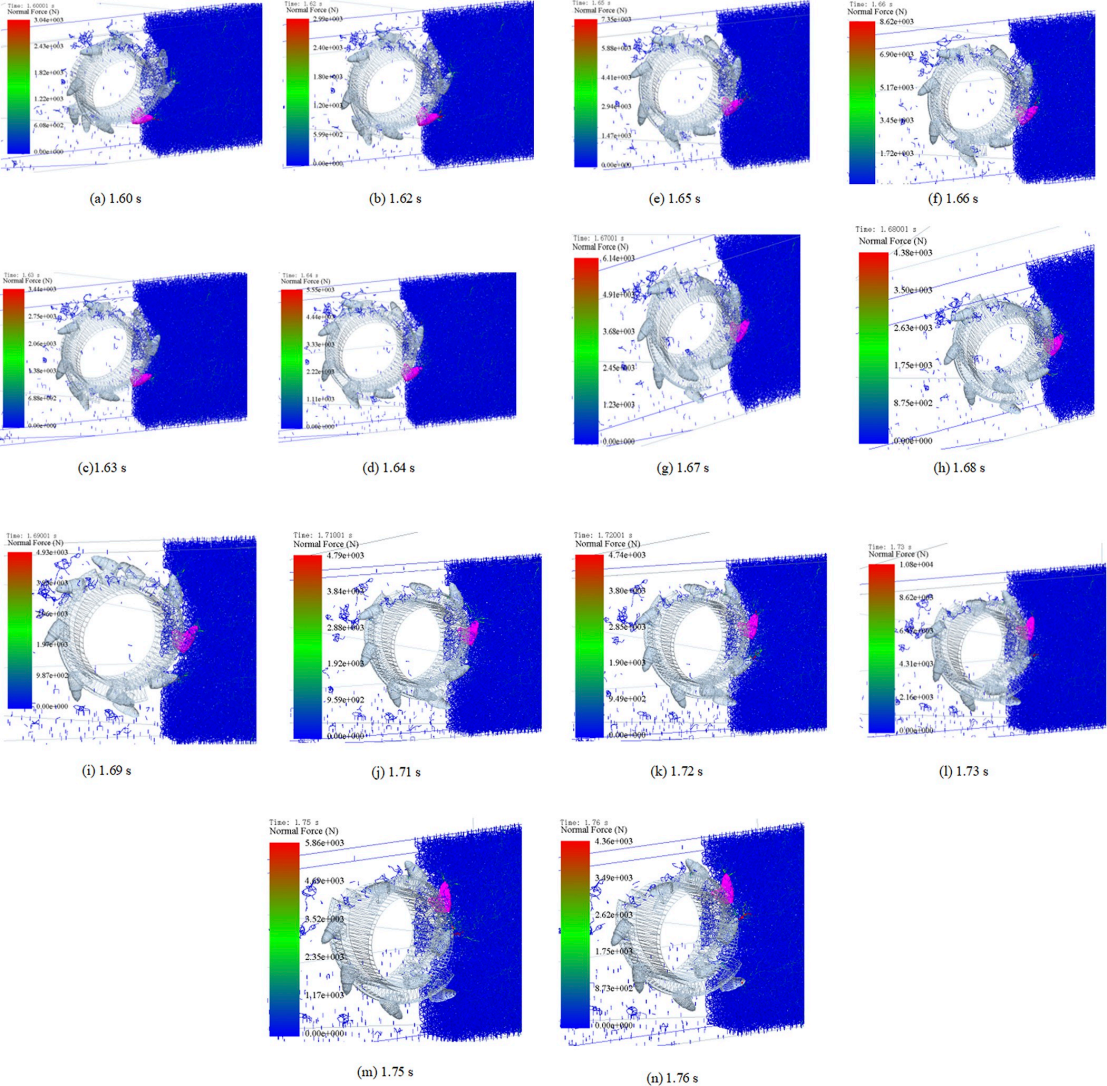
Distribution of force chains. (a)1.60 s. (b) 1.62 s. (c)1.63 s. (d) 1.64 s. (e) 1.65 s. (f) 1.66 s. (g) 1.67 s. (h) 1.68 s. (i)1.69 s. (j) 1.71 s. (k) 1.72 s. (l) 1.73 s. (m) 1.75 s. (n) 1.76 s.

The force chain of tooth cutting coal rock was obtained (Fig 5). In Fig 5(A), the tooth has just been cut into coal, and the coal in front of the tooth tip is subjected to the cutting force. The cutting teeth are further cut into coal, and the reactive force is distributed around the tooth tip, as shown in Fig 5(B). As the drum rotates, teeth cut off the coal; the main bulk of the force is located in the falling coal, and the coal body fractures, as shown in Fig 5(C). As the drum continues to rotate, the force of coal and rock appears above the pick, as shown in Fig 5(D). The teeth are further cut into the coal, and the coal rock is subjected to the largest force in front of the tooth tip, as shown in Fig 5(E).

The drum further rotates, and the force of coal and rock appears above the front cutter surface of the teeth, as shown in Fig 5(F). Further, the force appears above the front cutter surface of the cutter teeth, and its area increases, as shown in Fig 5(G). Teeth cut off coal, as shown in Fig 5(H) and 5(I), and the falling coal and the coal body fracture become the main force position. As the teeth cut the coal further, the coal force appears near the teeth’ tips and is distributed in an umbrella shape, as shown in Fig 5(J). Furthermore, the coal force appears around teeth in a radial distribution (Fig 5(K)) before finally appearing above the pick, as shown in Fig 5(L).

### 4.2 The statistical areas

**Fig 6:**
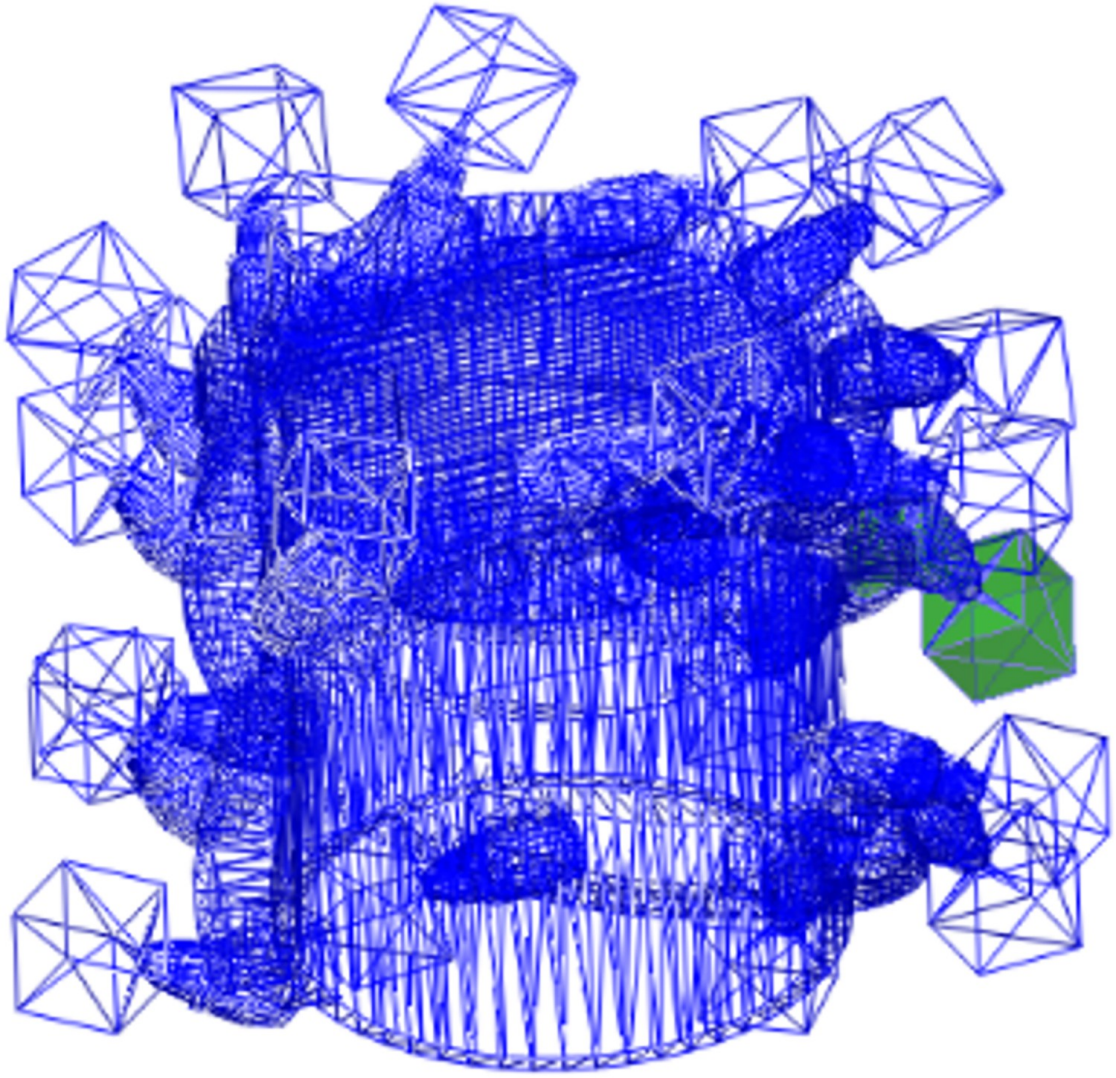
Drum and the statistical areas.

A total of 24 statistical areas were established to study the force of coal; each is located at the tips of the corresponding tooth and has a size of 100 mm × 100 mm × 100 mm. The drum and statistical areas are shown in Fig 6. All the areas move with the drum, meaning that the cutting force of the tooth acting on the coal can be determined in real time.

### 4.3 Force analysis in statistical areas

**Fig 7:**
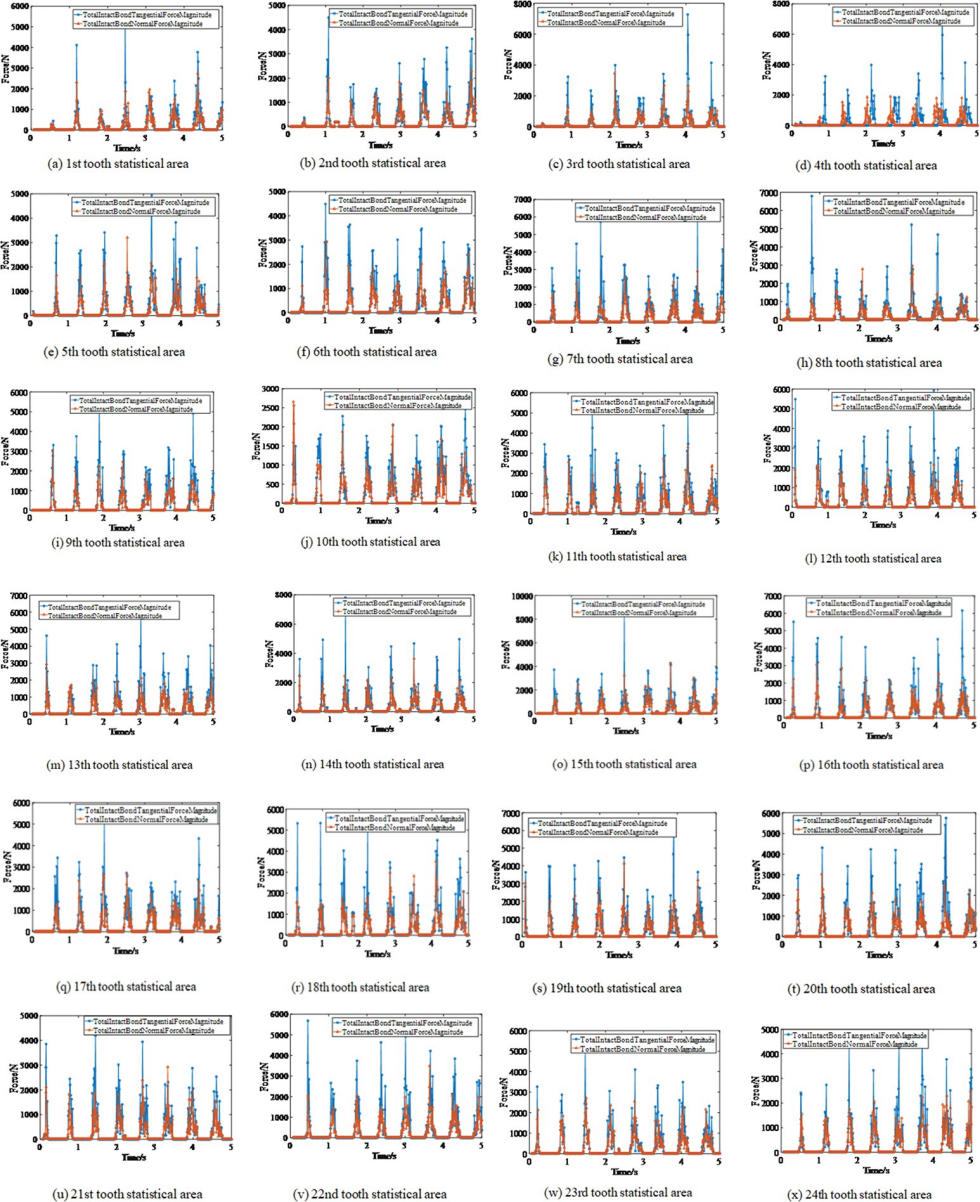
Normal and tangential forces in 24 statistical areas. (a) 1st tooth statistical area. (b) 2nd tooth statistical area. (c) 3rd tooth statistical area. (d) 4th tooth statistical area. (e) 5th tooth statistical area. (f) 6th tooth statistical area. (g) 7th tooth statistical area. (h) 8th tooth statistical area. (i) 9th tooth statistical area. (j) 10th tooth statistical area. (k) 11th tooth statistical area. (l) 12th tooth statistical area. (m) 13th tooth statistical area. (n) 14th tooth statistical area. (o) 15th tooth statistical area. (p) 16th tooth statistical area. (q) 17th tooth statistical area. (r) 18th tooth statistical area. (s) 19th tooth statistical area. (t) 20th tooth statistical area. (u) 21st tooth statistical area. (v) 22nd tooth statistical area. (w) 23rd tooth statistical area. (x) 24th tooth statistical area.

The tangential and normal forces acting on the coal in 24 statistical areas can be extracted to determine if the force that damages the coal rock damage is either normal or tangential, as shown in Fig 7. Fig 7 shows normal and tangential forces subjected to coal and rock in each statistical region.

According to the Hertz-Medlin model with bonding model theory, the normal force and the tangential force can break the bond when the force reaches the stress limit. Consequently, the coal particles are cut off from the coal wall. Therefore, the main reason for the bond fracture is that coal and rock are affected by both tangential force and normal force.

Fig 7 shows that the tangential and normal forces acting on the bond are not completely coincident. Even though their values are not generally equal, they sometimes differ.

Based on the simulation results, the average normal and tangential forces were obtained for 24 statistical areas, as shown in Table 4. Furthermore, as shown in Table 4, the ratio of the average tangential to the average normal force ranges between 1.34 and 1.79. The maximum ratio value occurred in the statistical area of the 11th pick in the first section. The minimum ratio was found in the statistical area of the eighth pick of the C section. Therefore, it was concluded that the bond failure is mainly caused by the tangential force, with the role of the normal force being secondary. This conclusion is consistent with references [7, 9, 10]. On the end plate, the four teeth breaking efficiency of Section A is the smallest, followed by Sections B, C, D, and E. The tooth-breaking efficiency on the blade is larger than that on the end plate, while the largest value appeared at the 12th tooth of the first transversal line.

**Table 4:** The force of coal rock in the statistical areas.

Statistical area	The average tangential Force/N	Maximum tangential force/N	The average normal force/N	Maximum normal force/N	Ratio of tangential force to normal force	The number of broken bonds
1st	387.7	5373	253.4	2739	1.53	6789
2nd	346.1	4497	233.5	2311	1.48	7736
3rd	412.5	7258	284.3	3466	1.45	8253
4th	366.4	7258	247.6	1902	1.48	7969
5th	402.7	4951	265.6	3209	1.52	8717
6th	336.5	4482	225	2939	1.5	8824
7th	405.7	6010	244.4	2905	1.66	10313
8th	217.9	6792	163.3	2974	1.33	10633
9th	262.7	5751	184.5	3003	1.42	10341
10th	276.3	2463	175.4	2658	1.58	10475
11th	276.3	5079	154.1	3300	1.79	11671
12th	289.3	5947	192.9	2258	1.5	13975
13th	373.5	6327	267.3	2943	1.4	12946
14th	367.9	7818	237.6	3645	1.55	13451
15th	374.1	8634	224.5	4231	1.67	12647
16th	315.3	6159	183.6	3060	1.72	13058
17th	380	5301	233.8	2663	1.63	12083
18th	267.1	5490	198.4	3501	1.35	12207
19th	415.6	6297	280.6	4130	1.48	12233
20th	416.6	5748	252.5	3023	1.65	12522
21st	405.1	4192	250	2935	1.62	12613
22nd	369.4	5682	258.3	3492	1.43	12771
23rd	399.7	5088	257.7	2711	1.55	12168
24th	360.7	4938	259.6	2475	1.39	11814

**Fig 8:**
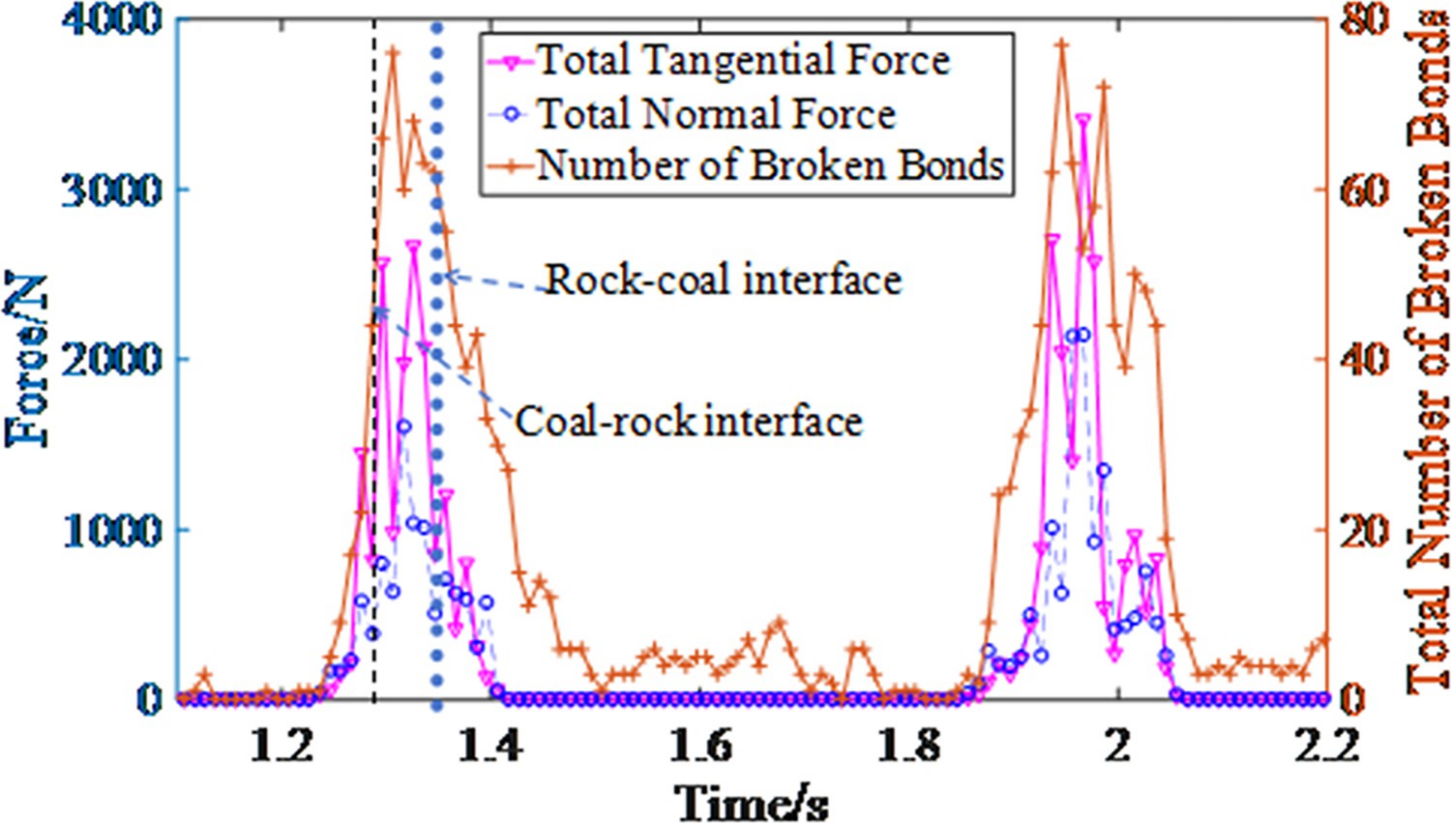
The relationship between the normal force, tangential force, and the number of broken bonds.

The 5th tooth was taken as an example. When coal and rock are damaged, the relationship between the normal and tangential force and the number of bonds in the statistical area is shown in Fig 8. The bond failure is caused by the combined action of the two. The bond failure number change trend is consistent with the tangential force but is not completely synchronous.

According to the simulation results, simulation process, and Fig 8 (time from 1.2 s to 1.4 s), when the tooth starts to cut the coal, the normal force on the coal varies between 173 N and 232 N. With further tooth cutting, the maximum normal force on the coal increases to 582 N. When the tooth cuts the coal-coal interface, the normal force on the coal-rock interface is 390.7 N. When the tooth cuts the rock, the maximum normal force on the rock is 1605 N. When the cutter cuts the rock-coal interface, the normal force on the rock is 512 N. Lastly, when the tooth cuts the coal, the normal force on the coal varies between 49 N and 710.9 N;

When the tooth starts to cut the coal, the tangential force on the coal varies between 61.4 N and 216 N. With further tooth cutting, the maximum tangential force on the coal increases to 1447 N. When the tooth cuts the coal-coal interface, the tangential force on the coal-rock interface is 802.2 N. When the cutter cuts the rock, the maximum tangential force on the rock is 2669 N. When the tooth cuts the rock-coal interface, the tangential force on the rock is 852 N. Lastly, when the tooth cuts the coal, the tangential force on the coal varies between 49 N and 1200 N.

Therefore, when the rock is in the middle of the coal seam, the force (including normal and tangential forces) on the rock is the highest. The force on the rock-coal interface is higher than that on the coal-rock interface.

### 4.4 Influence of drum rotation speed on the force of coal rock

**Fig 9:**
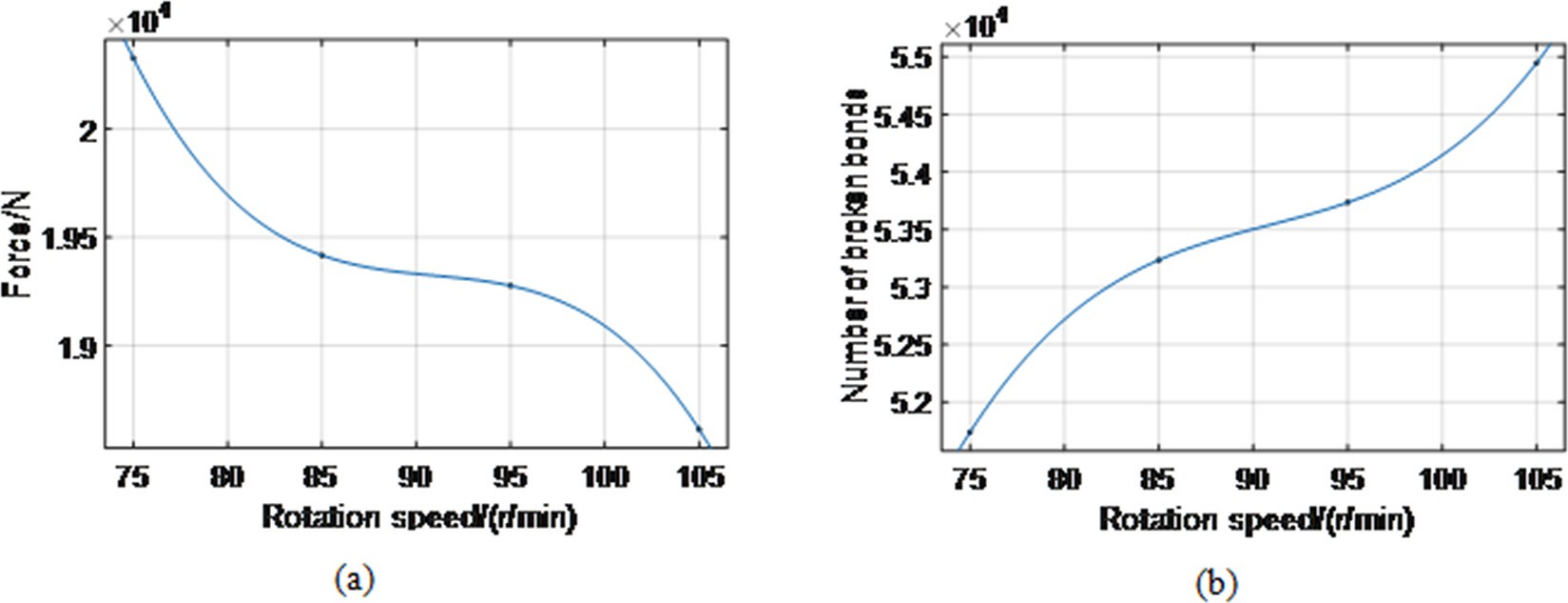
Relationship between the rotational speed and force on coal rock and the number of broken bonds. (a), (b).

Next, the influence of the drum rotation speed on the coal rock force was studied. In the simulation, the traction speed was set to 4 m/min while the drum rotation speeds were varied. Their values were 75 r/min, 85 r/min, 95 r/min, and 105 r/min. The simulation yielded average forces on coal rock of 20 326 N, 19 417 N, 19 276 N, and 18 613 N, with 51 740, 53 235, 53 735, and 54 947 broken bonds, respectively (see Fig 9).

As seen from Fig 9, with the increase in rotation speed, the force on coal rock decreases nonlinearly, and the number of broken bonds increases nonlinearly. When the traction speed is constant, the drum’s coal cut per unit time decreases with increased drum rotation speed. The total work done by the drum per unit time decreases, and correspondingly, the force acting on the coal rock by the drum decreases. Therefore, the force of coal and rock decreases with increased drum speed. The relationship between the drum’s rotation speed (*n*) and the force acting on the coal rock (*F*) is obtained by fitting, as shown in Formula (11). The cutting thickness becomes smaller with an increase in the drum’s rotation speed; the coal size decreases, and the dust increases. Consequently, the amount of coal the drum breaks per unit of time increases. Therefore, the number of broken bonds increases. Finally, the relationship between the drum rotation speed (*n*) and the number of broken coal rock bond particles (*N*) was obtained by fitting, as shown in Formula (12):

$$F(n)=-0.215\times n^{3}+58.66\times n^{2}-5344\times n+1.818\times 10^{5},$$ (11)

$$N(n)=0.2845\times n^{3}-77.52\times n^{2}+7084\times n-1.635\times 10^{5}.$$ (12)

### 4.5 Influence of traction velocity on the force of coal rock

**Fig 10:**
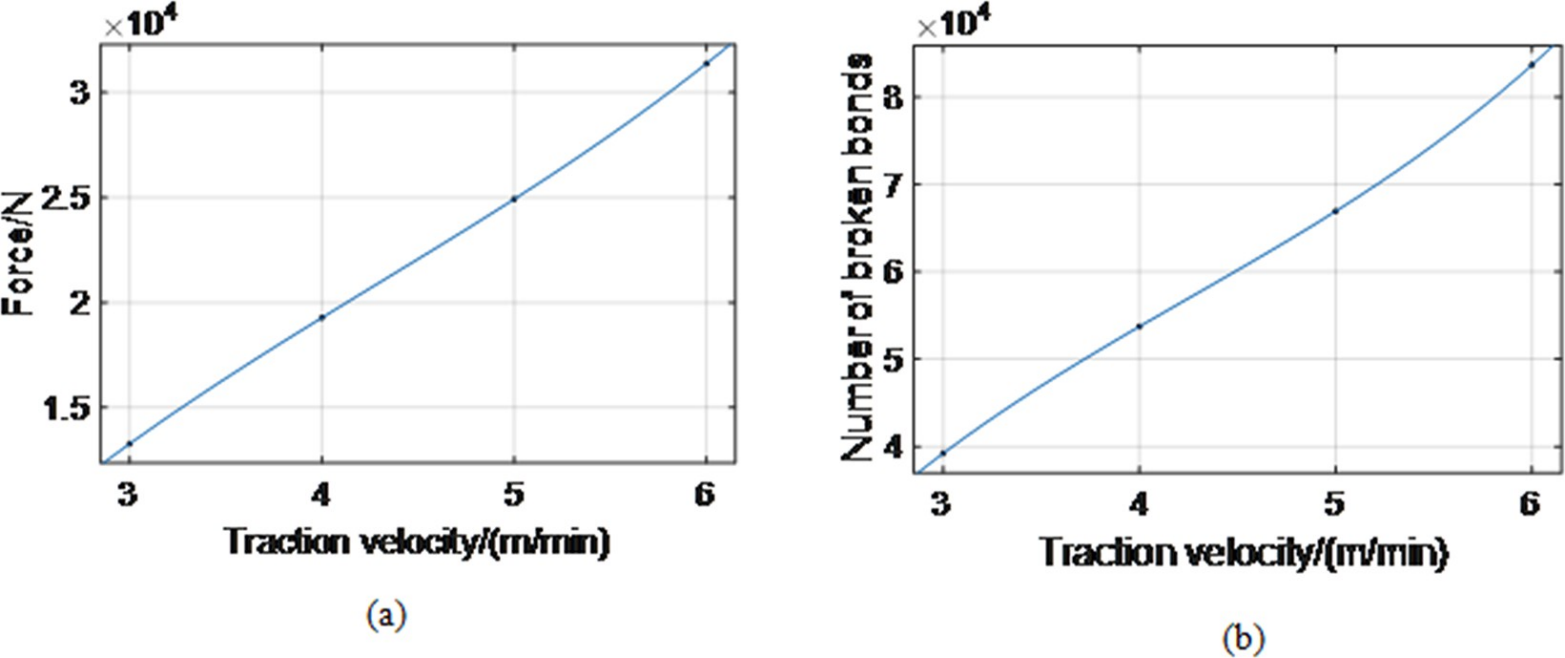
Relationship between the traction speed and force on coal rock and the number of broken bonds. (a), (b).

Aiming to study the effect of traction speed on coal and rock forces, the drum rotation speed was held constant at 95 r/min. In contrast, traction speeds were varied in the simulation. Their levels were 3 m/min, 4 m/min, 5 m/min, and 6 m/min. The average forces on coal and rock were 13 269 N, 19 276 N, 24 925 N, and 31 382 N, respectively, while the total numbers of broken bonds were 39 226, 53 735, 66 927, and 83 654, respectively (Fig 10).

According to Fig 10, the force on coal and rock increases approximately linearly with the traction speed. As the traction speed increased, the amount of coal cut by the drum per unit of time increased. The total work done by the drum per unit time increases, and correspondingly, the force acting on the coal rock by the drum increases. The coal-cutting amount of the drum in simulation time (5 s) increases with the drum’s traction speed. The number of coal failures also increased, meaning the number of broken bonds increased. Relationships between traction speed (*v*) and force of coal rock (*F*), and traction speed (*v*) and the number of broken bonds (*N*) were obtained by fitting:

$$F(v)=194.3\times v^{3}-2511\times v^{2}+16390\times v-18560,$$ (13)

$$N(v)=808.7\times v^{3}-10360\times v^{2}+57130\times v-60720.$$ (14)

### 4.6 Influence of tooth mounting angle on the coal rock force

**Fig 11:**
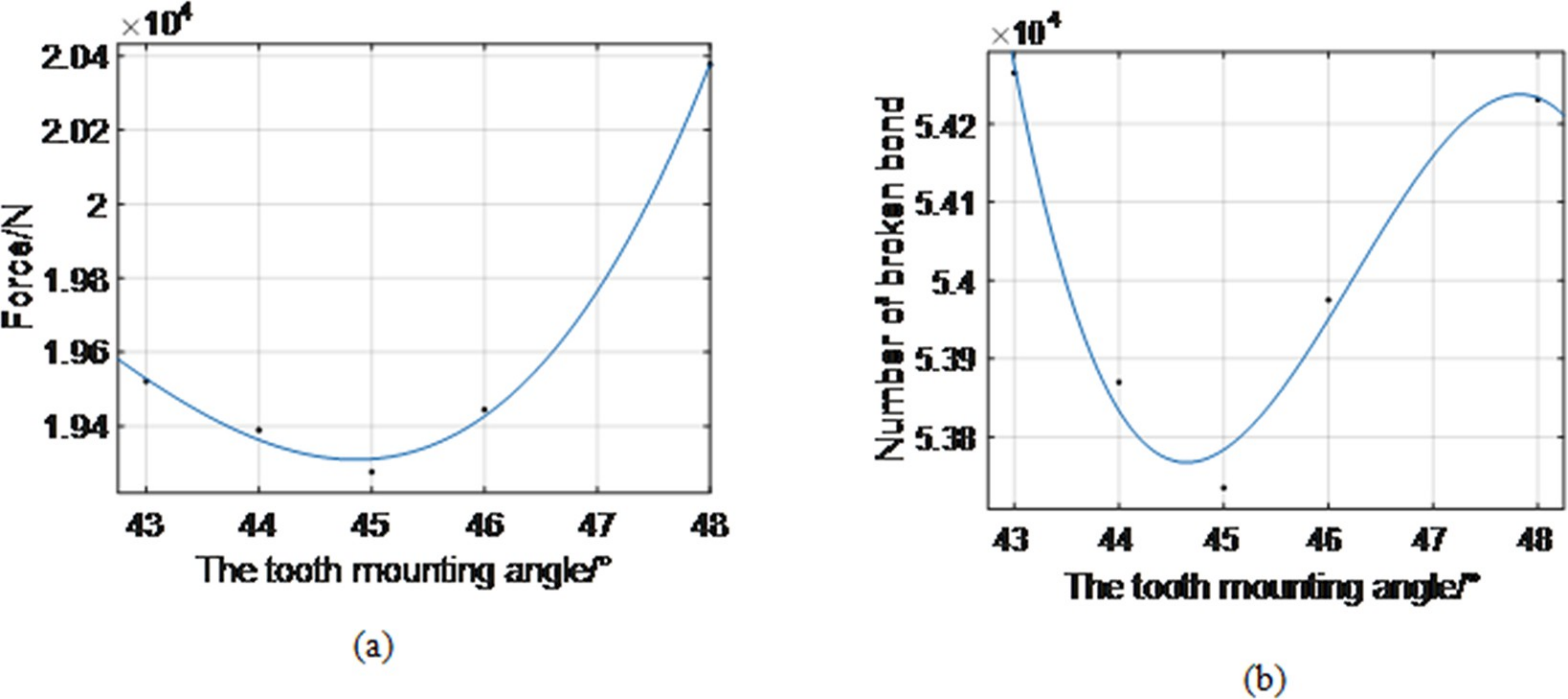
The relationship between the tooth mounting angle and the force acting on the coal rock and the number of broken bonds. (a), (b).

Further, the influence of tooth installation angle on the force of coal and rock was studied. The drum speed was constant, 95 r/min, and the traction speed (4 m/min). The tooth installation angles were varied and were 43°, 44°, 45°, 46°, and 48°, respectively. The average forces of coal and rock were obtained; they were 19 521 N, 19 389 N, 19 276 N, 19 444 N, and 20 377 N, respectively. There were 54 265, 53 870, 53 735, 53 975, and 54 231 bond failures, as shown in Fig 11.

The force acting on the coal and rock first decreases and then increases with the increase in the pick installation angle. The force of coal rock is minimum for the cutting angle of 45°. The number of bond failures first decreases and then increases once the mounting angle exceeds 45°. Relationships between the tooth installation angle (*α*) and the force of coal rock (*F*), and the tooth mounting angle (*α*) and the broken bonds number (*N*) are as follows:

$$F(\alpha )=9.079\times \alpha^{3}-1142\times \alpha^{2}+4.763\times 10^{4}\times \alpha -6.393\times 10^{5},$$ (15)

$$N(\alpha )=-29.19\times \alpha^{3}+4049\times \alpha^{2}-1.87\times 10^{5}\times \alpha +2.929\times 10^{6}.$$ (16)

## 5 Conclusions

In this paper, a model of coal containing gangue was established based on DEM. Moreover, a rigid-flexible coupled shearer section model was also established by RecurDyn. The drum cutting coal and rock process was simulated by discrete element and Recurdyn bidirectional coupling. The following conclusions are obtained:

1. The coal rock failure is caused by normal and tangential forces, where the tangential force plays a major role.
2. When the rock is in the middle of the coal seam, the force (including normal and tangential forces) on the rock is the largest; the force on the rock-coal interface is higher than that on the coal-rock interface.
3. The results have shown that the force acting on the coal rock decreases nonlinearly with the increase in the rotation speed. The number of broken bonds increases nonlinearly. When the rotating speed of the cylinder varies from 75 r/min to 105 r/min, the average coal force decreases from 20 326 N to 18 613 N; consequently, the number of bond failures increases from 51 740 to 54 947;
4. The force on coal rock increases nonlinearly as traction speed increases, while the number of broken bonds increases nonlinearly. When the traction speed increases from 3 m/min to 6 m/min, the average coal force increases from 13 269 N to 31 382 N; correspondingly, the number of bond failures increases from 39 226 to 83 654.
5. The force acting on the coal rock and the number of broken bonds first decrease and then increase as the tooth mounting angle increases. When the installation angle of the pick increases from 43° to 48°, the average load on coal decreases from 19 521 N to 19 276 N and then increases to 20 377 N. Furthermore, the number of bond failures decreased from 54 265 to 53 735 and then increased to 54 231. When the picking angle is 45°, coal’s average force and bond failure number are the smallest, i.e., 19 276 N and 53 735, respectively.

This study provides a reference for the research of coal breaking theory.
